# A2 Milk Enhances Dynamic Muscle Function Following Repeated Sprint Exercise, a Possible Ergogenic Aid for A1-Protein Intolerant Athletes?

**DOI:** 10.3390/nu9020094

**Published:** 2017-01-28

**Authors:** Ben Kirk, Jade Mitchell, Matthew Jackson, Farzad Amirabdollahian, Omid Alizadehkhaiyat, Tom Clifford

**Affiliations:** 1 School of Health Sciences, Liverpool Hope University, Hope Park, Liverpool L16 9JD, UK; jacksom1@hope.ac.uk (M.J.); amirabf@hope.ac.uk (F.A.); alizado@hope.ac.uk (O.A.); 2 Department of Sport, Exercise & Rehabilitation, Northumbria University, Newcastle Upon Tyne NE1 8ST, UK; j.mitchell@nutritionsociety.org (J.M.); tom.clifford@northumbria.ac.uk (T.C.)

**Keywords:** A2 milk, exercise recovery, muscle damage, team sports, muscle function

## Abstract

Hyperaminoacidemia following ingestion of cows-milk may stimulate muscle anabolism and attenuate exercise-induced muscle damage (EIMD). However, as dairy-intolerant athletes do not obtain the reported benefits from milk-based products, A2 milk may offer a suitable alternative as it lacks the A1-protein. This study aimed to determine the effect of A2 milk on recovery from a sports-specific muscle damage model. Twenty-one male team sport players were allocated to three independent groups: A2 milk (*n* = 7), regular milk (*n* = 7), and placebo (PLA) (*n* = 7). Immediately following muscle-damaging exercise, participants consumed either A2 milk, regular milk or PLA (500 mL each). Visual analogue scale (muscle soreness), maximal voluntary isometric contraction (MVIC), countermovement jump (CMJ) and 20-m sprint were measured prior to and 24, 48, and 72 h post EIMD. At 48 h post-EIMD, CMJ and 20-m sprint recovered quicker in A2 (33.4 ± 6.6 and 3.3 ± 0.1, respectively) and regular milk (33.1 ± 7.1 and 3.3 ± 0.3, respectively) vs. PLA (29.2 ± 3.6 and 3.6 ± 0.3, respectively) (*p* < 0.05). Relative to baseline, decrements in 48 h CMJ and 20-m sprint were minimised in A2 (by 7.2 and 5.1%, respectively) and regular milk (by 6.3 and 5.2%, respectively) vs. PLA. There was a trend for milk treatments to attenuate decrements in MVIC, however statistical significance was not reached (*p* = 0.069). Milk treatments had no apparent effect on muscle soreness (*p* = 0.152). Following muscle-damaging exercise, ingestion of 500 mL of A2 or regular milk can limit decrements in dynamic muscle function in male athletes, thus hastening recovery and improving subsequent performance. The findings propose A2 milk as an ergogenic aid following EIMD, and may offer an alternative to athletes intolerant to the A1 protein.

## 1. Introduction

Participation in unaccustomed eccentric exercise often causes exercise-induced muscle damage (EIMD) [1,2,3]. Physical activity with a large eccentric component includes resistance training, sprinting and plyometrics [4], all of which are common amongst sporting activities [5]. The exact causes of EIMD are ill-understood, but it is thought to be initiated by mechanical disruption of the myofibrils, which results in ultrastructural damage to the whole muscle cell [6,7]. It has been proposed that muscle function after EIMD is compromised through damage to the excitation-contraction (EC) coupling system, which is essential for muscle contraction and force output [8,9]. Mechanical damage coupled with the disruption of the EC coupling system is thought to activate a cascade of intracellular reactions such as an influx of calcium (Ca^2+^) into the cytosol that precipitate an acute phase inflammatory response [7]. In conjunction with other biochemical changes, such as increased reactive oxygen species (ROS) production [10], the inflammatory response might further degrade the muscle architecture in the days following muscle-damaging exercise [11,12]. Such changes can reduce the ability to generate power, evoke muscle pain (delayed onset muscle soreness, DOMS) and increase the release of intramuscular enzymes (i.e., creatine kinase) [6,13,14]. These symptoms generally peak 48 h after exercise [15] but can persist for 5–7 days [16,17]. This is particularly problematic in team-sports, where athletes are often required to train and/or compete on multiple occasions during a weekly cycle [18,19]. As a consequence, subsequent exercise performance might be impaired, and the propensity for injury increased [20]. 

Examples of the detrimental consequences of EIMD on exercise performance have been well documented [21]; twelve resistance-trained males demonstrated a 22.5% reduction in vertical jump height, and a 3-fold increase in muscle soreness after a bout of muscle-damaging drop jumps [22]. In a recent study with team-sports trained players, power generating ability (as measured by counter movement jump performance and reactive strength index) was reduced for up to 72 h after a single bout of repeated sprint exercise [1]. Such prolonged impairments in muscle function after muscle-damaging exercise are likely to negatively affect an athlete’s ability to perform in real-world sporting situations (i.e., competitions or training) [5,19]. 

Numerous studies have suggested that protein (PRO)-rich supplements are capable of attenuating indices of EIMD. The effects of whey PRO [23], casein PRO [24], leucine [25], and branched-chain amino acids (AA) [22] have been investigated for their ability to attenuate EIMD and hasten recovery, with many suggesting favourable effects [22,23,24].

More recently, a combination of PRO-carbohydrate (CHO) beverages such as dairy-milk [26], which have a favourable digestible and indispensable amino-acid score (DIAAS) > 1.0 [27], have expedited recovery of muscle function following eccentric-heavy exercise [28]. Dairy milk by mass contains ~80% casein and ~20% whey PRO, providing an advantageous balance of slow and fast digesting AA to the muscle for protein turnover [29]. By increasing AA availability, in particular leucine, milk activates anabolic signalling of mammalian target of rapamycin (mTOR), the key metabolic regulator for muscle PRO synthesis [30]. Post-exercise feeding of PRO and CHO can also favourably raise blood insulin levels from ~5 to 30 m/UL [31], providing an ideal insulinogenic environment which may suppress the breakdown of skeletal muscle via the ubiquitin proteasome pathway [28,32]. This increase in insulin action is attributed to increased microvascular perfusion [33].

In a series of studies [26,34,35,36] milk supplementation post-eccentric exercise was shown to attenuate symptoms of EIMD, such as muscle soreness and deficits in muscle function. At present, the mechanisms underlying reduction in EIMD with milk are unclear. However, one possible mechanistic explanation is that milk inhibits the activity of calpains and other degradative pathways upregulated after muscle-damaging exercise [37,38,39]. This, in turn, preserves the integrity of the muscle cell, especially the proteins responsible for force generation and transmission. Irrespective of the exact mechanisms, the above data suggest that milk is a promising recovery intervention after strenuous exercise [29]. However, for athletes who are intolerant to lactose and/or the A1 beta-casein PRO, found in regular milk, these ergogenic benefits remain elusive, including: PROs for muscle recovery, CHO for glycogen resynthesise, and electrolyte and vitamin replacement for re-hydration [40,41]. 

**Figure 1:**
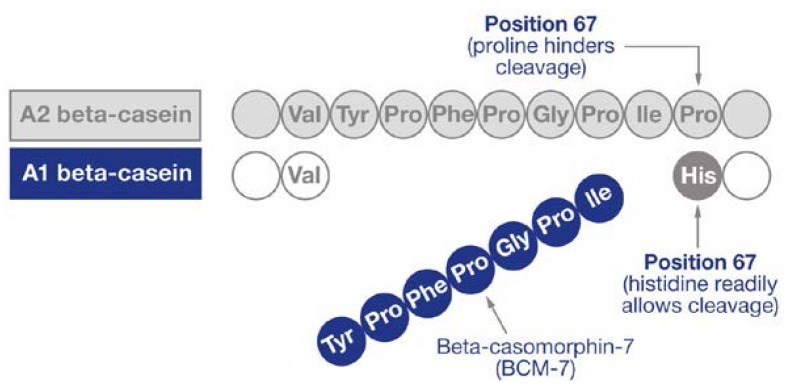
Structure of beta-casmorphin-7. Adapted from [48] (reproduced with permission).

The general assumption surrounding the milk intolerance syndrome is that it is triggered by lactose malabsorption [42,43]. However, as described in the 2010 National Institutes of Health Consensus statement on lactose intolerance, many who suffer from self-reported gastrointestinal (GI) issues display no evidence of insufficient lactase enzyme activity [44]. An alternative mechanism supported by growing evidence [45,46,47] is that bovine beta-cosmorphin-7 (BCM-7) (See Figure 1), derived from A1-beta casein in regular milk, but not A2-beta casein, expresses opioid receptors in the human GI tract upon digestion which may cause motility disorders, inflammation, abdominal pain and loose stools [44,48]. Furthermore, it has been speculated the interaction of lactose and BCM-7 with regular milk may exacerbate the above-mentioned symptoms [48]. Consequently, an alternative option for athletes who suffer from these GI discomforts with regular milk is warranted. A2 milk is a natural, biologically-occurring form of cow’s milk, identical in nutrient composition to regular milk, but lacks A1-beta casein and therefore BCM-7 expression, and as such may offer a substitute [49]. However, no study to date has investigated the effects of A2 milk on EIMD following strenuous exercise.

Accordingly, the aim of this study was to examine whether A2 milk is equally as effective as regular milk for attenuating EIMD, following a sport-specific bout of repeated sprint exercise. Hence, the authors hypothesised that A2 milk would be as effective as regular milk for attenuating markers of EIMD. In addition, both milk supplements would be more effective than a placebo.

## 2. Materials and Methods

### 2.1. Participants

Twenty-one healthy males, who regularly competed in team-sports (Gaelic football *n* = 7; soccer *n* = 7; rugby *n* = 7) were recruited to take part in this study (see Table 1). Sample size estimates were based on previous literature examining milk supplementation and EIMD that had shown a statistical effect [26,34]. Following institutional ethical approval (W14026049), all laboratory procedures, associated risks and benefits were illustrated both verbally and in written format, before participants provided written consent. Participants were familiarised with all laboratory procedures prior to study commencement and instructed to arrive at the laboratory in a rested state, having avoided strenuous physical activity for at least 72 h before testing. Participants were also instructed to refrain from exercise, nutritional supplements (whey PRO, casein PRO, branched-chain AA, creatine), caffeine, alcohol and anti-inflammatory drugs for the duration of the study. Participants were excluded if they had recently suffered from a musculoskeletal injury, were receiving prescribed medications or had an intolerance to dairy or lactose products. To minimise diurnal variation, participants were tested at the same time each day [50].

### 2.2. Study Design

**Figure 2:**
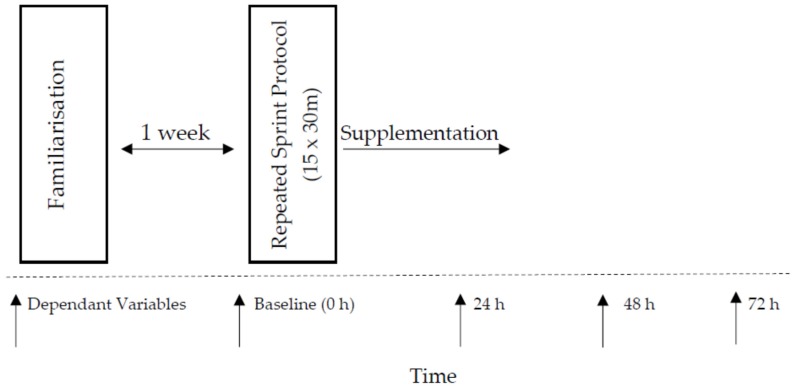
Experimental design of study protocol.

Participants were assigned to one of three independent groups, in a double-blind design. On the first day of the trial, participants performed a range of neuromuscular functional tests before the repeated sprint protocol. Immediately after the repeated sprint protocol, participants consumed their allocated drinks. The dependent variables (visual analogue scale, muscle soreness), countermovement jump height (CMJ), maximum voluntary isometric contraction (MVIC) and 20-m sprint were measured in the respective order prior to and 24, 48, and 72 h after the repeated sprint protocol (See Figure 2).

### 2.3. Nutritional Intervention and Dietary Control

Participants were randomly but equally divided according to their baseline MVIC score ascertained at familiarisation to consume one of three drinks: A2 milk (a2 semi-skimmed milk^TM^; The A2 Milk Company, Surrey, UK) *n* = 7; regular milk (Tesco semi-skimmed milk; Tesco Stores Ltd., Cheshunt, UK) *n* = 7; placebo (MyProtein maltodextrin 50 g mixed with water (MyProtein, Northwich, Cheshire, UK)) *n* = 7 (see Table 2). Each drink was prepared in masked bottles by an external staff member who was not involved in the trial to ensure both the researchers and participants were blinded from treatments. Participants recorded their food intake for 24 h prior to and for the 4 trial days through the use of food diaries. Instructions were given at familiarisation on how to correctly weigh food, measure liquids, and fill in the food diaries. Throughout the trial period participants were also instructed to verbally report any GI discomforts associated with the ingestion of milk supplements to the research team.

### 2.4. Baseline Performance Indices

#### 2.4.1. Visual Analogue Scale (Muscle Soreness)

Participants were required to squat down to an angle equal to 90° knee flexion with feet shoulder width apart and then return to the starting position. Participants then rated their perceived level of muscle soreness by marking a line on a scale between 0 mm (no pain) and 200 mm (unbearably painful). This was measured and recorded in mm for the pain score. See [1] for further description.

#### 2.4.2. Countermovement Jump (CMJ)

CMJ was assessed using a light timing system (Optojump, Microgate, Italy). Participants were instructed to squat down with their hands on their hips throughout and jump vertically. Participants were reminded that all efforts must be maximal. Three jumps with a 60-s rest in-between were performed with the peak jump height used for data analysis. See [39] for further description.

#### 2.4.3. Maximal Voluntary Isometric Contraction (MVIC)

MVIC force of the dominant knee extensor was recorded using a strain gauge (MIE Digital Myometer, MIE Medical Research Ltd., Leeds, UK). The knee joint angle was set before each contraction at 90° using a goniometer to minimise error derived from alteration in muscle length. All participants completed three isometric MVIC’s of 3-s duration, separated by a 60-s rest period. The peak MVIC from the three contractions was used for statistical analysis. See [1] for further description.

#### 2.4.4. 20 m Sprint Test

As described elsewhere [39], participants completed three maximal effort 20-m sprints each day on an indoor running track. Participants completed the sprint from a standing start 20 cm behind the line. Timing gates (Brower, Draper, UT, USA) were used to record sprint time with the fastest sprint used for analysis.

#### 2.4.5. Repeated Sprint Protocol

The repeated sprint protocol was based on previous studies [3,51] which successfully utilised it as a method of inducing muscle damage. A 30-m section of an indoor running track was marked using cones and timing gates. A further 10-m deceleration zone was marked at the end of the 30-m section. Participants first completed a warm up consisting of 400 m of self-paced jogging, and a series of dynamic sprint drills including high knees, heel flicks and walking lunges, which were conducted over a measured 10-m section of the aforementioned indoor running track. This was followed by a series of three practice sprints at the participants perceived 60, 80 and 100% of maximum speed. Following the warm up, participants were given 5 min to prepare themselves for the repeated-sprint protocol, during which time no static stretching was performed as it has been previously suggested to impair sprint performance [52]. Participants then completed 15 × 30 m sprints with a 60-s rest period in-between repetitions and were instructed to stop within the marked 10-m deceleration zone. Participants were reminded to exert maximal effort during each sprint. Strong verbal encouragement was given throughout.

### 2.5. Statistical Analysis

All data are expressed as mean ± standard deviations (SD). Participants food diaries were analysed for macronutrient content through dietary analysis software (Microdiet v2.5, Downlee systems Ltd., Salford, UK). Independent groups were analysed using a mixed model analysis of variance (SPSS Statistics 22, IBM, Chicago, IL, USA), with three group levels (A2 milk vs. regular milk vs. placebo) and four time levels (baseline, 24 h, 48 h, 72 h) to establish differences between groups. Mauchly’s test of sphericity was used to check homogeneity of variance for all variables; where necessary, any violations of the assumption were corrected using the Greenhouse–Geisser adjustment. Significant effects were followed up using Tukey post hoc analysis. The alpha level for statistical significance was set at *p* < 0.05 a priori. To estimate the magnitude of supplements effects, Cohen’s *d* effect sizes (ES) were calculated with the magnitude of effects considered: small (0.20–0.49), medium (0.50–0.79) or large (>0.80).

## 3. Results

### 3.1. Group Allocation

Analysis revealed no difference between groups in MVIC values used for group allocation (*p* = 0.79). Dietary analysis indicated there were no differences in energy (*p* = 0.82), carbohydrate (*p* = 0.48), protein (*p* = 0.44) or fat (*p* = 0.56) intake between the groups across the five days (*p* > 0.05; See Table 3).

### 3.2. Evidence of Muscle Damage

All measures of neuromuscular function (20-m sprint, CMJ height, MVIC, Muscle Soreness) showed main effects for time (*p* = 0.022, *p* ≤ 0.001, *p* ≤ 0.001, *p* ≤ 0.001, respectively) indicating the repeated-sprint protocol induced muscle damage.

### 3.3. Effects of Nutritional Supplement

#### 3.3.1. The 20-m Sprint

**Figure 3:**
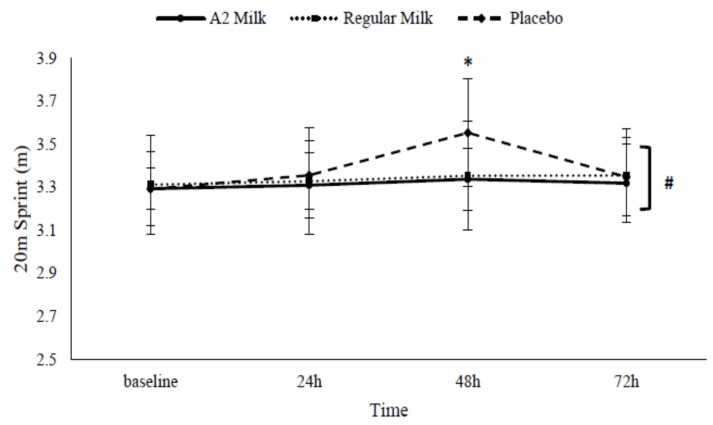
Change in 20-m sprint performance in response to exercise-induced muscle damage in the A2 milk (*n* = 7), regular milk (*n* = 7), and placebo (*n* = 7) groups. * Represents group–time interaction effect (*p* < 0.05). # Represents main effect for time (*p* < 0.05). Values presented as mean ± SD.

There were no group effects for the 20-m sprint (*p* = 0.743); however, a group–time interaction effect was present (*p* = 0.026). Post-hoc analysis demonstrated group differences at 48 h post EIMD in A2 milk vs. PLA (*p* = 0.046; ES = 1.09) and regular milk vs. PLA (*p* = 0.032; ES = 0.98). At 48 h, 20-m sprint time recovered quicker in A2 (3.3 ± 0.1) and regular milk (3.3 ± 0.3) vs. PLA (3.6 ± 0.3) (See Figure 3). Relative to baseline, decrements in 48 h 20-m sprint time were minimised in A2 (by 0.9 ± 3.4%) and regular milk (by 0.8 ± 1.2%) vs. PLA (by 6.0 ± 7.2%). 

#### 3.3.2. CMJ height

**Figure 4:**
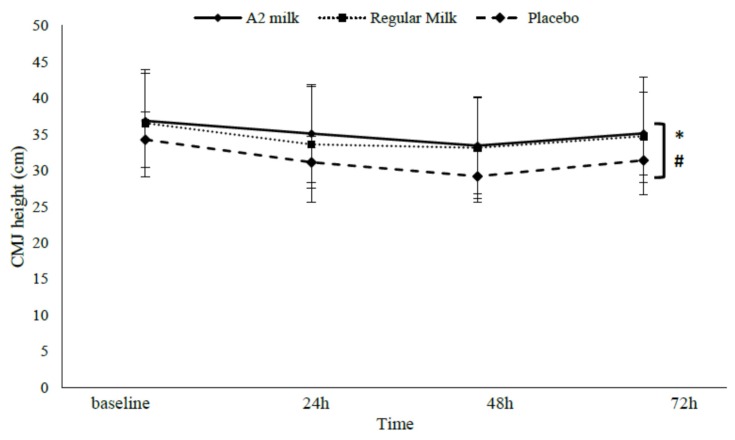
Change in countermovement jump (CMJ) height in response to exercise-induced muscle damage in the A2 milk (*n* = 7), regular milk (*n* = 7), and placebo (*n* = 7) groups. * Represents group difference (*p* < 0.05). # represents main effect for time (*p* < 0.05). Values presented as mean ± SD.

A group effect showed that CMJ height appeared to recover quicker in milk treatments vs. PLA (*p* = 0.041). Although no group–time interaction effects were present (*p* = 0.098), large effect sizes (1.42; 1.29) were again apparent at 48 h post EIMD, whereby CMJ recovered quicker in A2 (33.4 ± 6.6) and regular milk (33.1 ± 7.1) vs. PLA (29.2 ± 3.6), respectively (See Figure 4). Relative to baseline, decrements in 48 h CMJ height were minimised in A2 (by 1.6 ± 3.2%) and regular milk (by 2.5 ± 2.6%) vs. PLA (by 8.8 ± 4.4%).

#### 3.3.3. Maximal Voluntary Isometric Contraction (MVIC)

**Figure 5:**
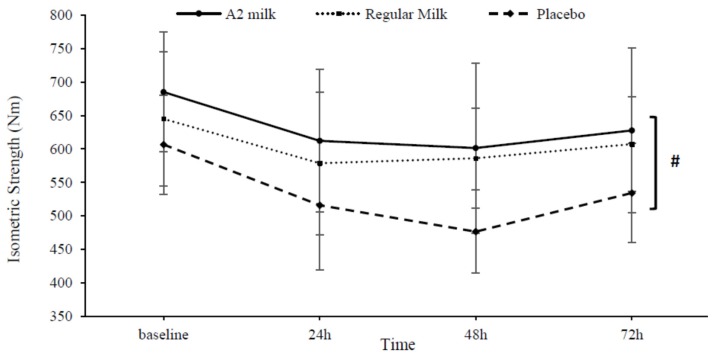
Change in maximal voluntary isometric contraction (MVIC) in response to exercise-induced muscle damage in the A2 milk (*n* = 7), regular milk (*n* = 7), and placebo (*n* = 7) groups. # Represents main effect for time (*p* < 0.05). No group or interaction effects were observed (*p* > 0.05). Values presented as mean ± SD.

No differences between groups (*p* = 0.151) or interaction effects between time and group (*p* = 0.069) were observed for MVIC (See Figure 5).

#### 3.3.4. Visual Analogue Scale (Muscle Soreness)

**Figure 6:**
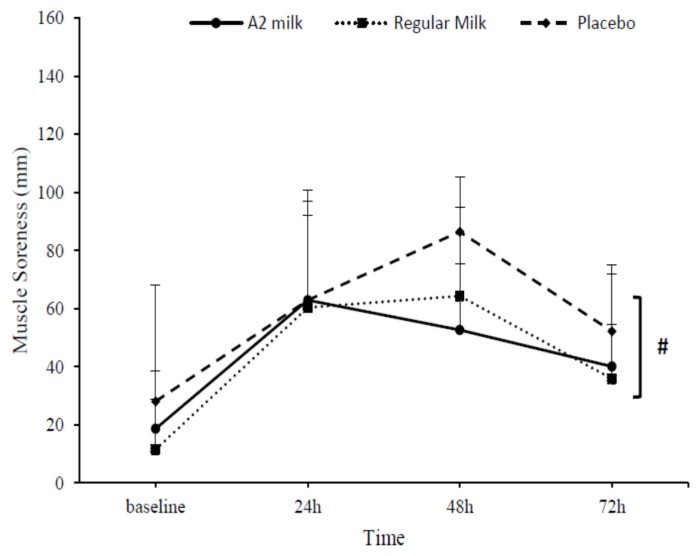
Change in perception of muscle soreness in response to exercise-induced muscle damage in the A2 milk (*n* = 7), regular milk (*n* = 7), and PLA (*n* = 7) groups. # Represents main effect for time (*p* < 0.05). No group or interaction effects present (*p* > 0.05). Values presented as mean ± SD.

No differences between groups (*p* = 0.490) or interaction effects between time and group (*p* = 0.152) were observed for muscle soreness (*p* > 0.05) (See Figure 6).

## 4. Discussion

This investigation sought to examine whether A2 milk was similarly effective to regular milk for attenuating EIMD. We found 500 mL of A2 or regular cow’s milk consumed immediately after the repeated sprint bout reduced decrements in 20-m sprint time (5.1 and 5.2%, respectively) and CMJ height (7.2 and 6.3%, respectively) 48 h after EIMD. Although statistical significance was not reached, MVIC was reduced to some degree with milk treatments (*p* = 0.069). These findings suggest A2 milk is an efficacious recovery beverage following EIMD, and perhaps, offers an alternative to athletes who experience GI discomfort with the expression of bovine BCM-7 derived from regular milk. 

The pertinent new finding from our data show there were no differences in measures of muscle function or soreness between milk treatments. Both products were isonitrogenous, containing the same proportions of PRO (~80% casein, ~20% whey), and identical amounts of CHO (24 g) and fat (9 g). In addition, the AA leucine concentration in milk treatments was similar (~1.7 g). Consequently, any effect on attenuating EIMD based on overall macronutrient ingestion would be similar. However, with regular milk there are documented negative effects of the bioactive peptide BCM-7 on GI motility [45,46], and interaction with lactose upon enzymatic digestion, which could theoretically slow absorption [48,53]. Considering this, perhaps A2 milk could offer an anabolic advantage for muscle recovery through enhanced digestion of PRO and absorption rates of AA to the muscle for protein synthesis. Nevertheless, our data show negligible effects with regard to which milk supplement was consumed, with both products similarly effective at alleviating symptoms of EIMD which was hypothesised by the authors. It may be interpreted the minor difference in PRO structure of A2 milk, is unlikely to influence its biological role in recovery from EIMD and offer an advantage to regular milk [49]. Although all participants were free from dairy intolerances prior to trial enrolment, it should be noted that compliance with milk products was satisfactory, with no verbal reporting of any discomforts. 

The benefit of A2/regular milk for male athletes corroborates previous research [34] which revealed similar improvements in muscle function 48 h after exercise, and additional studies examining the damage response after combined PRO–CHO supplementation [34,39,54,55,56,57]. Nevertheless, the aforementioned milk studies [26,39,54] employed a laboratory-based muscle damage protocol (i.e., isokinetic dynamometer or repeated drop jumps). To ensure our findings could be more readily translated to an athletic cohort, we induced muscle damage through a validated repeated sprint exercise protocol [3], to simulate some of the movements in team-sport competition. 

Several potential mechanisms may explain how A2/regular milk favourably attenuated losses in functional capacity after muscle-damaging exercise. In the present study, hyperaminacidemia following the digestion of whey/casein in milk may have initiated protein remodelling [29], primarily due to the presence of leucine [58], and the other DIAA crucial for a sustained anabolic response [59]. In addition, the synergistic intake of nutrients post-EIMD would conceivably mediate insulin release which may have suppressed exercise-induced proteolysis by dampening the activation of the ubiquitin proteasome pathway [29,60]. We propose that providing milk and its constituents during a period of catabolism preserved the muscle against adverse effects [61]. In turn, this may have protected contractile proteins responsible for force-generating capacity [39]. Reduced markers of pro-inflammatory cytokines in the days following EIMD have also been evident with milk supplementation [35,39]. Taken together, these mechanisms provide a feasible explanation as to how milk facilitated the maintenance of short-term dynamic muscle power output, as measured by CMJ-height and 20-m sprint performance, 48 h after exercise. In support, a plethora of research [28,38,62,63] has documented a well-established link between myofibrillar disruption and muscle functioning. Furthermore, efficacy of milk proteins to support greater anabolism with combined whey and casein has previously been shown in comparison to ingesting either isolated protein alone [64]. Interestingly, the benefits of milk on EIMD were not apparent until 48 h after exercise. This may be related to PRO degradation rates not increasing until 24 h after exercise [38]. Various muscle biopsy studies [65,66,67] have shown the symptoms of ultrastructural damage become progressively worse post-exercise, peaking at 48 h, and due to slow repair rate subside within 5–7 days [16,17].

Although milk treatments did not significantly attenuate losses of MVIC, there was a slight reduction at 48 h which supports existing data [22]. A possible explanation for the non-significance may be related to the differences in muscle actions during certain performance measures. For instance, assessing force production through CMJ-height and 20-m sprint speed involves eccentric-concentric muscle actions. In comparison, MVIC is measured through isometric strength production with no change in muscle length [53]. Similar reductions in loss of short-term isotonic power output following EIMD have been observed with beetroot juice ingestion, with no further beneficial effect on isometric strength [1,68]. Likewise, a single treatment of either cows or goats chocolate milk revealed no benefit in 2-h post-resistance exercise isometric strength assessed through mid-thigh pull performance [69]. Thus, perhaps a dynamic movement involving the stretch-shortening cycle is required to fully elucidate milk’s ability to attenuate EIMD, rather than a non-lengthening muscle contraction. Alternatively, the leucine dosage of ~1.7 g in the present study may have been suboptimal to maximise muscle protein synthesis, accounting for the non-significance. We cannot rule out this possibility considering the leucine threshold to maximise muscle remodelling post-exercise in young healthy athletes is recommended as ~2–3 g [53,70]. However, considering the positive effects milk treatments had on CMJ-height and 20-m sprint speed, this is difficult to conceive.

In line with previous research [22,35,71], muscle soreness peaked 48 h post-exercise in the present study. However, supplementation with A2 or regular milk had no beneficial effect on attenuating soreness, in contrast to other studies [35,39,72] which found soreness was reduced with milk in males. Interpreting this finding is difficult as the exact mechanism responsible for DOMS is still unknown [72]. It has been suggested the cause is due to damage to the endomysium surrounding muscle fascicles that occurs following the biphasic assault of exercise. In turn, this may result in nerve ending potentiation which has shown to correlate with increased muscle soreness [14,39]. However, milk’s effects on this mechanism is yet to be elucidated. We postulate the milk dosages administered in the present study (~18 g PRO in 500 mL), which is slightly less than that reported in [35,72], might have been inadequate to maximise the post-exercise PRO metabolism response [70,73] associated with perceptions of soreness.

There are some limitations in this study that need to be acknowledged. Firstly, no muscle biopsy or inflammatory blood marker was obtained to measure the mechanisms related to muscle damage and therefore we cannot directly infer how milk treatment enhanced recovery. Secondly, it is unclear if individuals who are indeed A1-protein intolerant will display the same recovery benefits with A2-milk following EIMD, as all participants reported no milk allergies prior to trial commencement. Future trials should consider these aspects and include direct measures of GI symptoms.

## 5. Conclusions

The primary implication from our study supports the use of A2 milk for athlete recovery in sports nutrition settings. We found the consumption of 500 mL of A2 or regular milk following repeated sprint exercise can limit decrements in muscle function in male team-sports athletes, thus hastening recovery. From a practical perspective, these findings suggest that A2 milk might offer an alternative to athletes who experience GI issues with regular milk [45]. However, this possibility needs to be investigated in athletes who are A1 beta-casein intolerant.

## Figures and Tables

**Table 1:** Group subject characteristics.

Group	Baseline MVIC (N·m)	Age (Years)	Height (m)	Weight (kg)
A2 milk	685 ± 89	23 ± 3	178.8 ± 5.1	79.4 ± 10.1
Regular Milk	644 ± 74	23 ± 1	183.0 ± 8.6	81.4 ± 13.1
Placebo	606 ± 74	22 ± 1	180.7 ± 5.5	77.1 ± 7.8

Values presented as mean ± standard deviations (SD; *n* = 7 per group). MVIC: maximal voluntary isometric contraction. No significant differences were detected between groups for any variable (*p* > 0.05).

**Table 2:** Macronutrient content per 500 mL of supplement. PRO: protein; CHO: carbohydrate.

Group	Energy	PRO	CHO	Fat
A2 milk	1005 kJ/235 kcal	18 g	24 g	9 g
Regular milk	1046 kJ/250 kcal	18 g	24 g	9 g
Placebo	920.5 kJ/220 kcal	0 g	50 g	0 g

**Table 3:** Mean energy and macronutrient intake for the three supplement groups over the five trial days (*p* > 0.05).

Group	Energy	PRO	CHO	Fat
A2 milk	11,984 kcal	510 g	1604 g	452 g
Regular milk	11,879 kcal	571 g	1390 g	419 g
Placebo	11,451 kcal	584 g	1403 g	420 g
